# Pore-scale effects during the transition from capillary- to viscosity-dominated flow dynamics within microfluidic porous-like domains

**DOI:** 10.1038/s41598-021-83065-8

**Published:** 2021-02-16

**Authors:** A. Yiotis, N. K. Karadimitriou, I. Zarikos, H. Steeb

**Affiliations:** 1 School of Mineral Resources Engineering, Technical University of Crete, Chania, Greece; 2 Institute of Mechanics (CE), University of Stuttgart, Stuttgart, Germany; 3 Environmental Research Laboratory, National Center for Scientific Research ‘Demokritos’, Agia Paraskevi, Greece; 4 Stuttgart Center for Simulation Technology, University of Stuttgart, Stuttgart, Germany

**Keywords:** Environmental impact, Chemical engineering, Hydrology

## Abstract

We perform a numerical and experimental study of immiscible two-phase flows within predominantly 2D transparent PDMS microfluidic domains with disordered pillar-like obstacles, that effectively serve as artificial porous structures. Using a high sensitivity pressure sensor at the flow inlet, we capture experimentally the pressure dynamics under fixed flow rate conditions as the fluid–fluid interface advances within the porous domain, while also monitoring the corresponding phase distribution patterns using optical microscopy. Our experimental study covers 4 orders of magnitude with respect to the injection flow rate and highlights the characteristics of immiscible displacement processes during the transition from the capillarity-controlled interface displacement regime at lower flow rates, where the pores are invaded sequentially in the form of Haines jumps, to the viscosity-dominated regime, where multiple pores are invaded simultaneously. In the capillary regime, we recover a clear correlation between the recorded inlet pressure and the pore-throat diameter invaded by the interface that follows the Young–Laplace equation, while during the transition to the viscous regime such a correlation is no longer evident due to multiple pore-throats being invaded simultaneously (but also due to significant viscous pressure drop along the inlet and outlet channels, that effectively mask capillary effects). The performed experimental study serves for the validation of a robust Level-Set model capable of explicitly tracking interfacial dynamics at sub-pore scale resolutions under identical flow conditions. The numerical model is validated against both well-established theoretical flow models, that account for the effects of viscous and capillary forces on interfacial dynamics, and the experimental results obtained using the developed microfluidic setup over a wide range of capillary numbers. Our results show that the proposed numerical model recovers very well the experimentally observed flow dynamics in terms of phase distribution patterns and inlet pressures, but also the effects of viscous flow on the apparent (i.e. dynamic) contact angles in the vicinity of the pore walls. For the first time in the literature, this work clearly shows that the proposed numerical approach has an undoubtable strong potential to simulate multiphase flow in porous domains over a wide range of Capillary numbers.

## Introduction

Multiphase flow in macroporous media (i.e. typical pore sizes greater than 50 nm) is a ubiquitous process of significant scientific and technological interest. It is encountered in a series of energy-related engineering applications, including those exploitation of natural resources (e.g. geothermal energy^1,2^, Secondary and Enhanced Oil recovery^3–5^), but also in environmental-related ones, including soil remediation from persistent anthropogenic pollutants^6,7^, the sustainable management of groundwater aquifers, and geologic carbon sequestration as a climate change mitigation strategy^8,9^. Such processes are characterized by the flow of immiscible fluids within the heterogeneous and anisotropic internal structure of geologic porous media over large spatial scales (ranging from a few meters to hundreds of kilometres), which is dominated by the complex interplay between capillary, gravity, and viscous forces acting simultaneously upon moving interfaces. These interactions, that originally emanate at the scale of individual pores and fractures (i.e. micrometers to centimetres), are responsible for the intrinsically highly non-linear field-scale behaviour of the immiscible flow process in the longer term (e.g. at time scales ranging from days to years), leading to different flow patterns and sweeping efficiency in recovery applications^10–12^.

The established modelling approach for the description of such processes on the application (macro) scale relies on reduced order models, where the porous medium is treated as an effective continuum, and fluxes (momentum, mass or energy) are related to volume-averaged variables through phenomenological transport expressions. The underlying assumption implemented in this approach is that transport and compositional properties can be adequately defined at a Representative Elementary Volume (REV) scale, with typical dimensions that may be several orders of magnitude larger than the actual pore sizes (e.g meters at the REV-scale compared to 10–500 $\mu m$ at the actual pore-scale). At this scale, flow and phase transport are conveniently modelled through generalized forms of Darcy’s equation extended to multiple phases (e.g. Forchheimer^13^, Brinkmann^14^). All transport properties of the medium pertaining to individual phases are expressed as lumped parameters, volume-averaged at the REV-scale (e.g. relative permeability, dispersivity, thermal conductivity), and are taken to be functions of local phase saturations only (and more recently also of specific fluid–fluid interfaces^15^. Such parameters are assumed to account for all non-linear effects emanating at the pore-scale and are typically calculated using core flooding experiments under quasi-static flow conditions^16,17^.

In recent years, significant physical insight on the actual dynamics of interfaces during immiscible two-phase flows through porous media has been provided experimentally, by the use predominantly of transparent 2D micromodels. The development of these artificial porous media prototypes has been a turning point in the study of multiphase flows in macro-porous materials, as they allowed for the first time a direct visual observation of complex pore-scale flow regimes that otherwise occur in real non-transparent 3D structures. Such models have progressed from simpler glass bead packings, with an effectively random internal geometry (but tunable porosity and average pore size)^18,19^, to structures with well-defined internal geometry constructed using chemical^20^, plasma^21^ or laser etching^22^, stereo lithography, optical lithography^23,24^, and more recently soft lithography^25,26^ and mechanical milling^27^.

Being effectively microfluidic devices, the developement of such micromodels also followed relevant technological progress both in the fields of high-precision flow and pressure control allowing for the design of elaborate experimental study of immiscible flows in well-defined porous structures under a very wide range of flow conditions. Experimental studies using such micromodels revealed the highly non-linear interaction between interfacial forces and the emergence of quite different flow regimes ranging from stable (piston-like) to fractal-like displacement patterns in the form of viscous and capillary fingers^19,28–30^. They were used to study flow regimes and their contribution to effective relative permeability curves in immiscible flows^31–33^,the effect of viscosity on sweeping efficiency^34^, the correlation between capillary pressure and interfacial area^35^, and more recently flows involving disconnected phases in the form of ganglia^27,36–38^ that diverging significantly from the established Darcian flow description.

Such experimental investigations have been complemented by elaborate pore-scale numerical models, starting with the pioneering work of Fatt^39^ that relied on the simplified representation of the void space as a regular network of interconnected pores and throats. Recent advances in computational power combined with novel parallel programming approaches has allowed the simulation of immiscible flows at sub-pore-scale resolutions. This approach offers a more rigorous method to study pore-scale physics under a wide variety of flow conditions, at the expense, however, of significant computational resources and time. Such approaches include highly parallel Lattice Boltzmann methods^40–44^, which are however limited to relative small density differences and a restricted choice of boundary conditions to generate flow, Particle-Hydrodynamics (SPH)^45–47^, the Volume-of-Fluid (VOF) method^48,49^, Level-Set, and Phase Field approaches^50,51^. Such contributions have offered significant insight on pore-scale physics that determine the temporal evolution of interfaces under a wide range of flow conditions. However, the verification of such numerical tools against well-designed microfluidic experiments still remains quite limited^45,52^.

In this contribution, we develop a microfluidic setup that allows for the simultaneous measurement of the inlet pressure during immiscible displacement within 2D PDMS porous-like domains at extremely high accuracy (i.e. $\pm 20 {\rm Pa}$ which is sufficient for resolving both capillary and viscous pressure drop) and the corresponding phase distribution patterns (and interface movement) using optical microscopy. This allows for the direct correlation between forces acting upon the interface and the pore size that it transverses at any given time and the quantification of viscous and capillary pressure drop at the pore-scale under precisely controlled flow conditions. In parallel, we employ a robust pore-scale level-set simulator to study interfacial dynamics and inlet pressure dynamics under comparable flow conditions. By performing a series of validation tests against well-established theoretical flow models, we verify both the accuracy of our experimental and the efficiency of numerical simulator to recover flow dynamics under both capillary and viscous-dominated flow conditions.

## Materials and methods

### Experimental microfluidic setup

**Figure 1:**
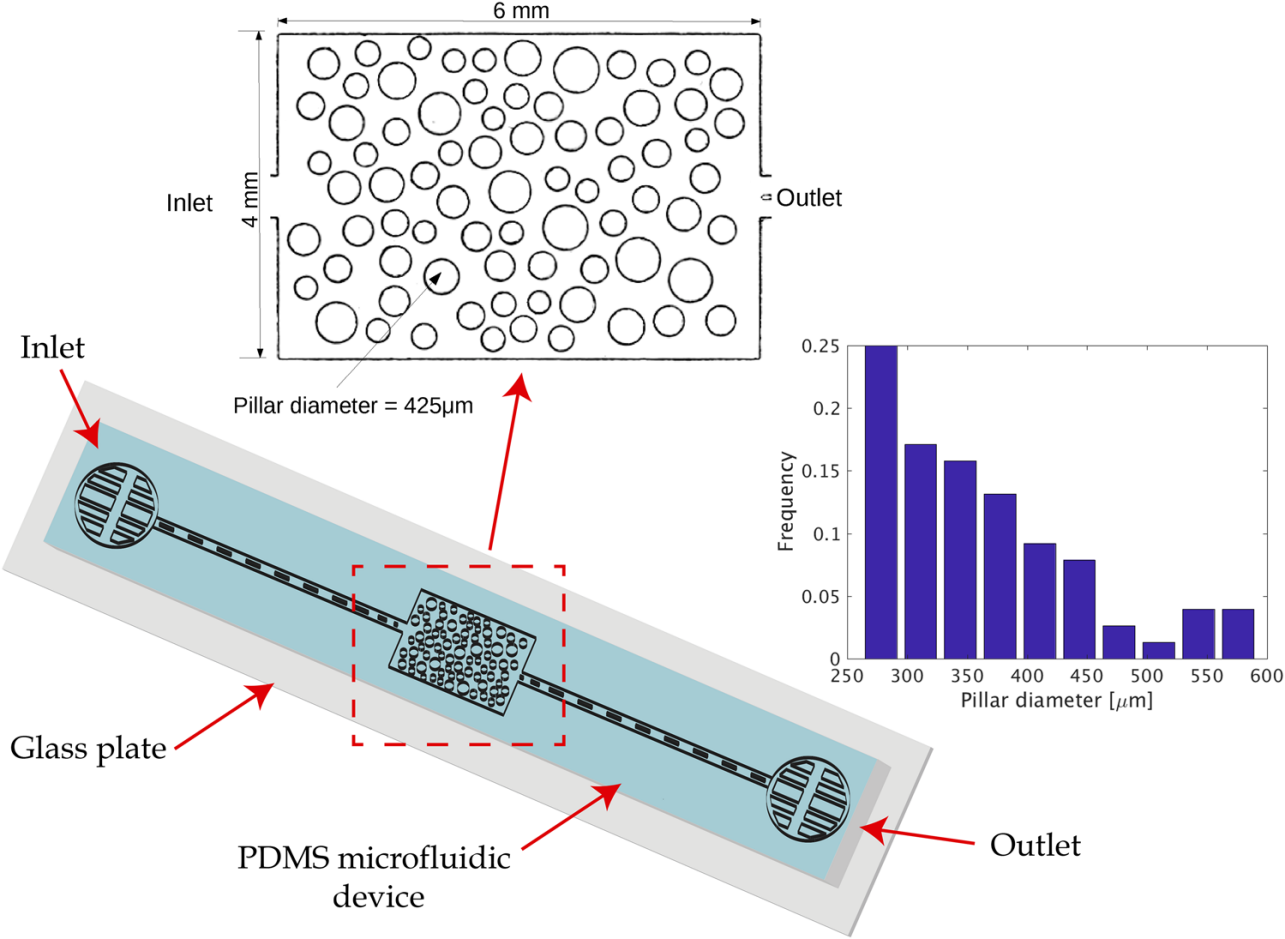
Schematic of the microfluidic domain used in this study. The inset shows the the central area (6 mm x 4 mm x 115 $\mu m$) of the micromodel that contains circular pillars and effectively serves as a porous domain and the size distribution of the pillar diameters.

In this study, we have developed a porous-like micromodel made of Polydimethylsiloxane (PDMS) (Dow Corning Sylgard by Sigma Aldrich) using a standard soft lithography methodology^25,53–56^ As shown in Fig. 1 (top), the micromodel consists of a central rectangular area 6 mm long and 4 mm wide and two flow channels (inlet and outlet) both 13mm long and 500 $\mu m$ wide. The flow channels are connected to two liquid buffers that serve for the control of fluid injection and recovery. The entire micromodel is 115 $\mu m$ deep and the rectangular central area contains 76 non-overlapping cylindrical pillars (that appear as circles in Fig. 1 (left) with diameters selected from a random size distribution in the range of 275 and 575 $\mu m$ (average diameter of 380 $\mu m$) as shown in Fig. 1 (right). The minimum distance between adjacent pillars is at least 1/3 the depth of the micromodel with a resulting porosity of the domain equal to 0.62.

The inlet liquid buffer is connected to a microfluidic syringe pump (neMESYS 1000N by CETONI) through Polytetrafluoroethylene (PTFE) capillary tubing with an internal radius of *R* = 0.5 mm. A microfluidic pressure sensor (MPS0 by ElveFlow) with a maximum pressure reading of 7000 Pa and typical precision of 20 Pa is fixed at the PTFE tubing, approximately 10 cm from the inlet buffer of the microfluidic device. The sensor readings are digitally recorded on a PC at a maximum sampling rate of 10 Hz with the use of a 16 bit ADC Beckhoff programmable logic controller by Cetoni adapted to a Cetoni Base 120, in parallel with the neMESYS 1000N syringe pump. The pump is also controlled by a PC using the software QMixElements by Cetoni. The outlet buffer is directly open to the environment (i.e. at atmospheric pressure).

**Figure 2:**
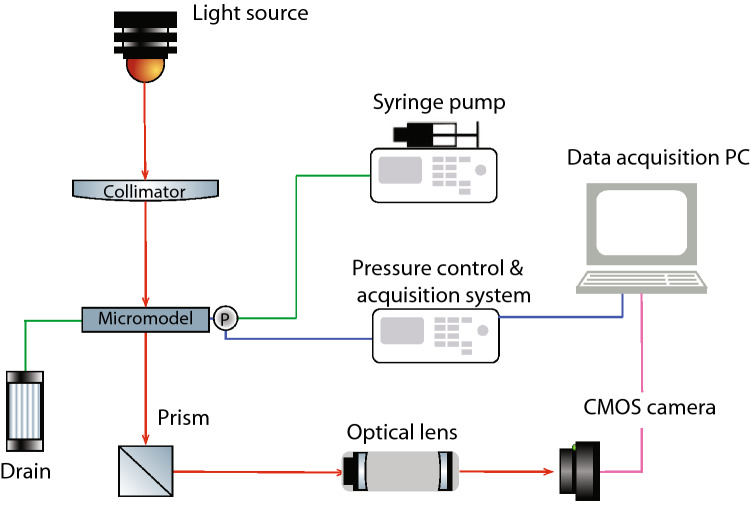
Schematic of the experimental setup used in this study to record the inlet pressure and capture the corresponding phase distribution patterns, simultaneously, during immiscible flow within the micromodel. The micromodel is placed above a fixed glass surface located between the light source and the prism.

An ‘open-air’ optical microscope is used to record the phase distribution patterns during the experiments. It consists of a LED light source emitting at 590 nm attached to an objective lens (Canon F 3.2/135 mm), which is used to focus a collimated beam of light at the top side of the micromodel. The micromodel is positioned on top of a transparent glass plate with a cubical prism (Edmund Optics) of size 50 mm is located just below. This serves for diverting the lighted micromodel image horizontally towards a telephoto lens (Sigma 135 mm F1.8 DG HSM), where it is magnified and recorded by a CMOS camera with a 5 Mpx sensor and a pixel size of 3.45 $\mu m$ (Allied Vision, GigE GC-D-2450), shown in Figure 2. The camera is capable of recording images at maximum rate of 15 fps directly to a PC through an Ethernet card. The entire setup is placed on a vibration-free optical table (Standa Ltd).

In all our experiments, 3M Fluorinert oil (FC-43 by Sigma-Aldrich) is used as the wetting phase (w-phase), while water dyed with ink (Talens Ecoline 578) serves as the invading non-wetting phase (nw-phase). The viscosity and density of Fluorinert (according to the supplier) is $\mu _o$ = 4.7 mPa s and $\rho _o$ = 1860 kg/m$^3$, respectively, while for the dyed water we take $\mu _{w}$=1.0 mPa s and $\rho _{w}$ = 1000 kg/m$^3$, respectively. The interfacial tension between Fluorinert and water is taken to be $\gamma$ = 55 mN/m, while the wetting angle is determined experimentally, as discussed below.

### Numerical model

The immiscible two-phase flow of Fluorinert and water in the previously described microfluidic domain is also studied numerically using the conservative Level-Set method proposed by Olsson et al.^57,58^, which is implemented using the commercially-available software platform Comsol Multiphysics v5.4. The method relies on the solution of the incompressible formulation of the Navier–Stokes equation;

1 $$\begin{aligned} \rho \left( \frac{\partial {\vec {u}}}{\partial {t}}+ \vec {u} \cdot \nabla \vec {u} \right) =\nabla \cdot \left( -p{\mathbf {I}}+\mu \left( \nabla \vec {u}+\left( {\nabla {u}}\right) ^T\right) \right) +\vec {F}_s \end{aligned}$$

the mass conservation equation;

2 $$\begin{aligned} \nabla \cdot \vec {u}=0 \end{aligned}$$

and the identity tensor;

3 $$\begin{aligned} {\mathbf {I}}=\delta _{ij}\vec {e}_{i} \otimes \vec {e_{j}} \end{aligned}$$

where $\rho \left( \vec {x},t\right)$ is the phase density (either Fluorinert or water), $\vec {u}\left( \vec {x},t\right)$ is the velocity vector, $p\left( \vec {x},t\right)$ is the local pressure, $\mu \left( \vec {x},t\right)$ is the phase viscosity, $\vec {F}_s\left( \vec {x},t\right)$ is the interfacial tension vector that develops only at the narrow fluid–fluid interfacial region and ${\mathbf {I}}$ is 2nd order identity tensor with the Kronecker delta. The Navier–Stokes equations (1,2), are coupled with an equation describing the advection of the interface under the velocity field, $\vec {u}\left( \vec {x},t\right)$;

4 $$\begin{aligned} \frac{\partial \phi }{\partial {t}}+\vec {u}\cdot \nabla \phi =\lambda \nabla \cdot \left( \epsilon \nabla \phi -\phi (1-\phi )\frac{\nabla \phi }{\left| \nabla \phi \right| }\right) \end{aligned}$$

where $\phi \left( \vec {x},t\right)$ is an auxiliary Level-Set variable that varies smoothly from zero to unity within the interfacial region (i.e. $0\le \phi \le 1$) and takes the predesignated extreme value within either of the two bulk phases (either $\phi =0$ or $\phi =1$), $\lambda$ is the so called re-initialization parameter and $\epsilon$ is a parameter that controls the interface thickness through the ‘numerical’ diffusion of $\phi$. Equation (4) is used to calculate the advection (flow) of the interface position between the two fluids under the effect of the velocity field $\vec {u}$ from Eq. (1). It is also used for the calculation of local interfacial forces $\vec {F}_s=\gamma \kappa \vec {n}_i= -\gamma \left( \nabla \cdot \frac{\nabla \phi }{\left| \nabla \phi \right| }\right) \nabla \phi$, where $\gamma$ is the interfacial tension, $\kappa =-\nabla \cdot \vec {n}_i$ is the local interface curvature and $\vec {n}_i=\frac{\nabla \phi }{\left| \nabla \phi \right| }$ is the unit vector normal to the interface.

The phase density and viscosity is calculated as a function of $\phi$ as follows;

5 $$\begin{aligned} \rho =\rho _{w}+\left( \rho _o-\rho _{w}\right) \phi \end{aligned}$$

and

6 $$\begin{aligned} \mu =\mu _{w}+\left( \mu _o-\mu _{w}\right) \phi \end{aligned}$$

Obviously, the no slip wall condition at fluid-solid interfaces (commonly used for single phase flows) would fail here to describe the movement of liquid interfaces tangentially to the solid walls of the domain. For this reason, we assume a viscous slip boundary condition^59^, where the fluid adjacent to the walls is allowed to move with a tangential velocity equal to

7 $$\begin{aligned} u_{\tau }=\frac{\lambda _s}{\mu }\sigma _{\tau } \end{aligned}$$

where $\lambda _s$ is the length and $\sigma _{\tau }$ is the shear stress in the direction parallel to the wall, calculated in the fluid side of the wall using Newton’s Law of viscosity as $\sigma _{\tau }=-\mu \frac{\partial {u_{\tau }}}{\partial n}$. $\lambda _s$ is a parameter related to the roughness of the walls and actually denotes the distance from the external side of the wall where the fluid velocity is taken equal to zero. Therefore, Eq. (7) relates the tangential interface velocity to its gradient normal to the wall. Thus, the interfacial force at the solid walls is modified as follows to account for both the slip velocity and the wettability of the interface;

8 $$\begin{aligned} \vec {F}_s=\gamma \delta \left( \vec {n}_w \cdot \vec {n}_i-cos(\theta _0)\right) \vec {n}_i-\frac{\mu }{\lambda _s}\vec {u}_{\tau } \end{aligned}$$

where $\vec {n}_w$ is the unit vector normal to the wall, $\vec {u}_{\tau }$ is the interface velocity vector tangentially to the wall, $\theta _0$ is the equilibrium wetting angle and $\delta$ is a smooth Dirac $\delta$-function at the position of the interface, taken here as $\delta =6 \left| \nabla \phi \right| \left| \phi (1-\phi ) \right|$.

### Validation of numerical model and experimental setup

We first perform a series of numerical simulations and experimental measurements in order to verify both the accuracy of our pressure sensors, but also the ability of the Level-Set model to simulate accurately viscous and capillary pressure drop in a wider range of flow injection rates. For this purpose, we have selected two flow scenaria in a circular capillary with well-established analytical and/or numerical solutions. The immiscible displacement process (either drainage or imbibition) in the following sections is typically described by two dimensionless parameters; The Capillary number, $Ca_w$, which denotes the ratio of viscous over capillary forces at the pore scale, $Ca_w=\frac{\mu _w u}{\gamma cos(\theta _0)}$, and the Viscosity ratio of the ‘invading’ over the ‘defending’ fluid, $M=\frac{\mu _{w}}{\mu _o}$. Here, *u* is the interstitial (pore-scale) velocity of the liquid.

#### Immiscible flow in a single capillary at low Ca values

**Figure 3:**
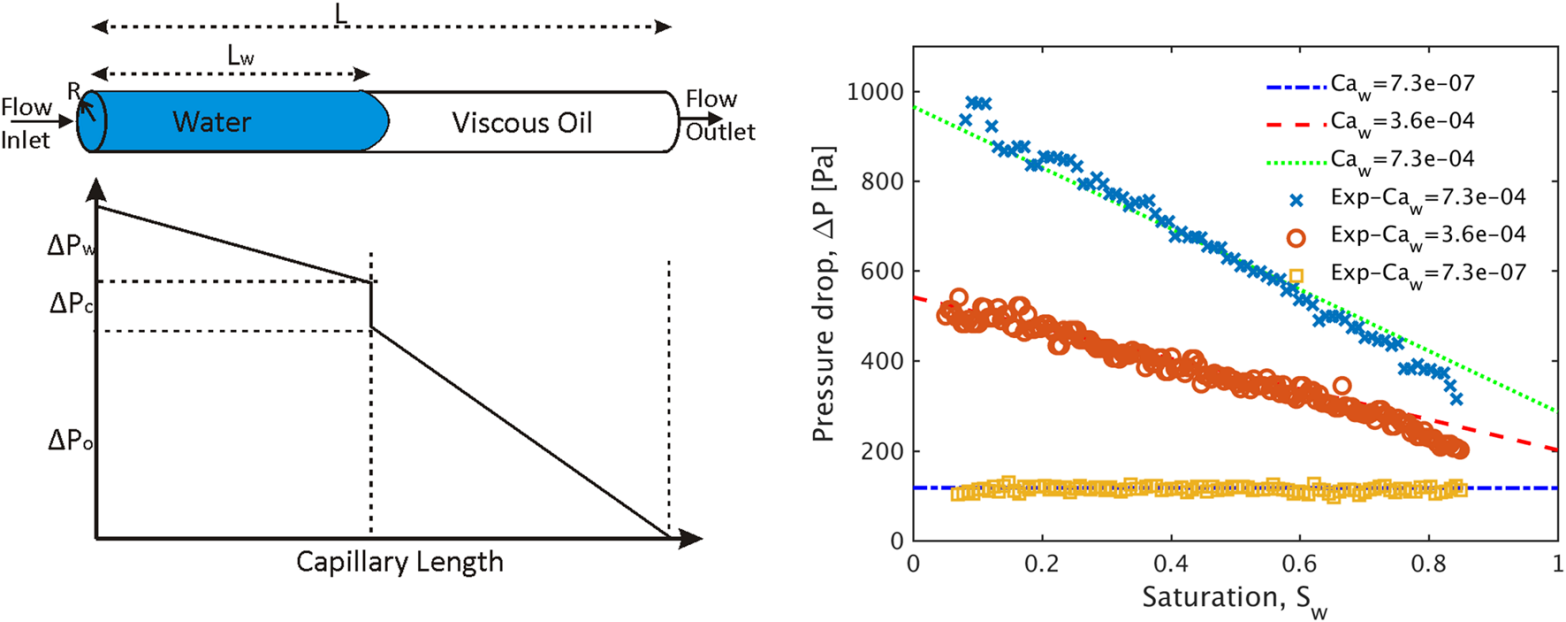
(Left) Schematic of the single capillary immiscible flow experiment described in “Immiscible flow in a single capillary at low Ca values” and the corresponding pressure drop along the capillary (for a given position of the fluid–fluid interface, i.e. fixed value of $S_w$). The total pressure drop $\Delta P$ across the capillary consists of two viscous terms (i.e. $\Delta P_o$ for the more viscous w-phase, $\Delta P_{w}$ for the less viscous w-phase) and a capillary pressure term $P_c$. (Right) Measured pressured drop across the capillary as a function of $S_w$ for 3 different values of $Ca_w$ (shown as discrete data points). The straight lines correspond to the solution of the Washburn equation for the same values of $Ca_w$.

We consider a horizontal PTFE capillary of total length *L* = 0.22 m and internal radius *R* = 0.5 mm (i.e. $L/R\gg 1)$, which is initially fully saturated by the more viscous w-phase (i.e. Fluorinert). The less viscous nw-phase (i.e. water) is then injected from one side of the capillary at a constant flow rate, *q*, while the other end is kept at ambient pressure, as shown in Fig. 3 (left). During this drainage experiment, the pressure sensor is fixed exactly at the inlet of the tube and the inlet pressure is recorded at a rate of 1–10Hz (depending on the flow rate) until the aqueous nw-phase fully displaces the wetting one and reaches the outlet of the capillary. The saturation of water, $S_w$, vs time is monitored both optically (by recording the length of the tube occupied by water, $L_w$, over the total length, i.e. $S_w=L_w/L$), but also by considering the injected liquid volume (measured by the syringe pump software) over the overall internal volume of the capillary, i.e. $S_w=\frac{\Delta V_w}{\pi R^2 L}$.

The total pressure drop across the capillary, $\Delta P$, can be then expressed as a function of $S_w$, following the approach of Washburn^60^ in the limit where $Ca_w\ll 1$. In this case, $\Delta P(S_w)$ is due to the combined effects of the viscous pressure drop within both phases plus a fixed contribution due to the capillary pressure drop, $\Delta P_c$, at the fluid–fluid interface as shown in Fig. 3a. The viscous pressure drop within each phase (i.e. the bulk nw- and w-phase regions across the interface) can be then approximated using Poiseuille’s equation for laminar flow;

9 $$\begin{aligned} \Delta P_i= \frac{8q\mu _i}{\pi R^4 } L_i \end{aligned}$$

where $i=w,o$ and $L_i$ is the length of the capillary occupied by each phase. The latter is measured from the inlet until the current position of the interface for the invading nw-phase and from the interface until the outlet of the capillary for the receding Fluorinert phase. The capillary pressure across the interface can be calculated from the Young-Laplace equation;

10 $$\begin{aligned} \Delta P_c=\frac{2\gamma cos(\theta _0)}{R} \end{aligned}$$

Thus the total pressure drop can be expressed as a function of $S_w$ as follows;

11 $$\begin{aligned} p(S_w)=\frac{\Delta P(S_w)}{\Delta P_{c}} = \frac{\Delta P_w+\Delta P_o+\Delta P_c}{\Delta P_{c}}= 4 \frac{Ca_w}{\xi }\left( \frac{\mu _w-\mu _o}{\mu _w}S_w +\frac{\mu _o}{\mu _w}\right) +1 \end{aligned}$$

where $$\xi =r/L$$.

In the above equation, we can distinguish two extreme cases;

1. When $Ca_w/\xi \ll 1$ *then* $\Delta P/\Delta P_{c} \approx 1$, namely the total pressure drop is independent of $S_w$ and practically equal to the capillary pressure $\Delta P_c$,
2. Otherwise, when $Ca_w/\xi \gg 1$ *then* $\frac{\Delta P(S_w)}{\Delta P_{c}} = 4 \frac{Ca_w}{\xi }\left( \frac{\mu _w-\mu _o}{\mu _w}S_w +\frac{\mu _o}{\mu _w}\right)$, a condition where viscous pressure drop is dominant at a length-scale of L.

Based on these considerations, we performed a series of immiscible flow experiments within the above configuration by injecting water at flow rates in the range of $10^{-4}\le q \le 1$ ml/min, effectively corresponding to $Ca_w$ values in the range of $7.3\times 10^{-8}\le Ca_w \le 7.3\times 10^{-4}$, in order to verify the accuracy our pressure sensor under both flow conditions. We should note that due to the dimensions of the capillary, the displacement experiments correspond to a range $3.6\times 10^{-5}\le \frac{Ca_w}{\xi } \le 3.6$.

Figure 3 (right) shows the recorded pressure data at the inlet of the capillary for three different values of $Ca_w$. As expected $\Delta P$ is a monotonically decreasing linear function of $S_w$ due to the displacement of the more viscous w-phase (i.e. Fluorinert) by the injected less viscous aqueous nw-phase. We observe that in the limit of very low values of $Ca_w$, the pressure drop is practically constant with $S_w$, due to a negligible contribution of viscosity to the overall pressure drop at very low flow rates. For $Ca=7.3\times 10^{-7}$ the average pressure drop over $S_w$ for is found equal to $\Delta$ P = 117 ± 20 Pa, which corresponds to a contact angle $\theta _0=58\pm 6^\circ$ at the fluid–fluid interface (as derived from Eq. 10). Based on this average contact angle, the expected theoretical behaviour based on the above Washburn formulation (i.e. Eq.11) is also plotted as a function of $S_w$ in Fig. 3 (right), and is found to be in very good agreement with the recorded experimental data over 3 orders of magnitude of $Ca_w$. This analysis demonstrates the excellent sensitivity of the pressure sensor and its suitability for the microfluidic experiments that will be discussed in the following sections.

#### Dynamic contact angle in a single capillary

In the previous section, we have shown both experimentally and analytically that the total pressure drop along a long circular capillary can be adequately described by assuming a constant pressure drop across the interface (besides the viscous pressure drop within the bulk liquid phases). Obviously, this is merely an approximation based on the assumption that the apparent wetting angle (and the corresponding radius of curvature) of the interface is practically constant and independent of $Ca_w$. Such an assumption is valid at lower $Ca_w$ values where viscous forces in the vicinity of the interface (i.e. at a length scale comparable to the pore diameter) are negligible compared to capillary ones. During however immiscible flow in actual porous domains, the interstitial (pore-scale) velocity of the interface changes abruptly as it moves through wider pore-bodies and adjacent narrower pore-throats (a phenomenon known as Haines jumps). When these jumps occur, the local (pore-scale) interface velocity may increase several orders magnitude, resulting in non-negligible viscous flow effects that may alter the apparent contact angle, $\theta _0$ of the interface.

**Figure 4:**
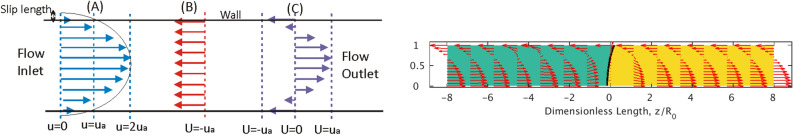
(Left) Schematic of the bulk phase (away from the fluid–fluid interface) radial velocity profile within the capillary for an immobile frame of reference **(A)**, and for a frame of reference that moves in the positive flow direction with velocity, $u_a$, similar to the average fluid velocity **(C)**. (Right) Steady-state phase distribution and velocity vector field for the single capillary imbibition problem from the solution of the coupled Level-Set/Navier–Stokes equations in an axisymmetric computational domain for $Ca_w=10^{-2}$ and $\theta _0=20$°. The w-phase is shown on the left side of the figure in green colour.

In order to verify the ability of our numerical Level-Set model to accurately recover equilibrium and dynamic contact angle dynamics over a wider range of (larger) $Ca_w$ values relevant to our study, we performed a series of numerical simulations focusing on a short section of the capillary close to the interface. Here we assume that this section serves as a proxy for an actual pore (i.e. $L \rightarrow r$, compared to $L \gg r$ in the previous section). Following the approach of Sheng and Zhou^59^, we consider a wetting fluid injected at a constant flow rate $q=\pi R^2 u_a$ in a capillary with a circular cross-section of radius *R* and length $L=8R$ (where $u_a$ is the average flow velocity). The interface is initially located midway along the length of the capillary and is taken flat at the beginning of each simulation. In order to allow for sufficient time for the interface to assume its steady-state curvature, while remaining within our simulation window, we impose a moving wall boundary condition in the opposite direction of the expected interface movement (here due to imbibition), as shown in Fig. 4 (left). The velocity of the capillary walls are thus taken equal to $u_w=-u_a$, while we impose a fully developed Poiseuille flow profile on both sides of the capillary, as $u(r)=2u_a\left( \frac{R^2-r^2}{R^2} \right) -u_a$. This approach is equivalent to a frame of reference that moves along with the fluid–fluid interface with its average velocity in the positive flow direction, while the walls of our capillary move in the negative flow direction with respect to this moving frame of reference. This formulation allows for the study of interfacial dynamics over a very wide range of $Ca_w$ values (several orders of magnitude) without the need to simulate a very large computational domain with a fixed frame of reference with respect to the walls. At the capillary walls we apply again a viscous slip boundary condition where the fluid velocity next to the wall is haines

12 $$\begin{aligned} u_{fx}=\frac{\lambda _s}{\mu }\sigma _{xr}-u_a \end{aligned}$$

where $\lambda _s$ is the slip length, that denotes the actual distance from the external side of the wall where the fluid velocity is taken equal to zero.

Figure 4 (right) shows the steady-state velocity field calculated for the above problem for $\theta _0=20^\circ$ and $Ca_w=10^{-2}$. The solution is calculated assuming axial symmetry of the flow around the x-axis. As discussed also above, this velocity field corresponds to a frame of reference moving with the same average velocity as the fluid. Therefore at steady-state the interface remains immobile with respect to this frame, while the radial velocity distribution takes positive values in the centre of the pillar and negative closer to the walls.

An extensive numerical study of the effect of the $Ca_w$ value on the shape of the interface reveals that its curvature progressively changes for 1/R at very low $Ca_w$ values (a limit that corresponds to the equilibrium contact angle, $\theta _0$, of the previous section) towards larger radii as $Ca_w$ increases (see Fig. 4 left). At $Ca_w=2\times 10^{-2}$ the radius becomes practically infinite and the interface flat. This effectively corresponds to a $90^\circ$ contact angle, much larger than the equilibrium $20^\circ$ angle shown here for $Ca_w=5\times 10^{-5}$. By increasing the Ca value even more, the concavity of the fluid–fluid interface changes towards the negative flow direction, effectively corresponding to contact angles higher than $90^\circ$. We can define the apparent contact angle by merely geometric parameters as follows;

13 $$\begin{aligned} \theta =tan^{-1}\left[ \frac{1-h^2}{2h}\right] \end{aligned}$$

where h is the dimensionless distance of the interface at r = 0 from the z = 0 plane at steady-state.

**Figure 5:**
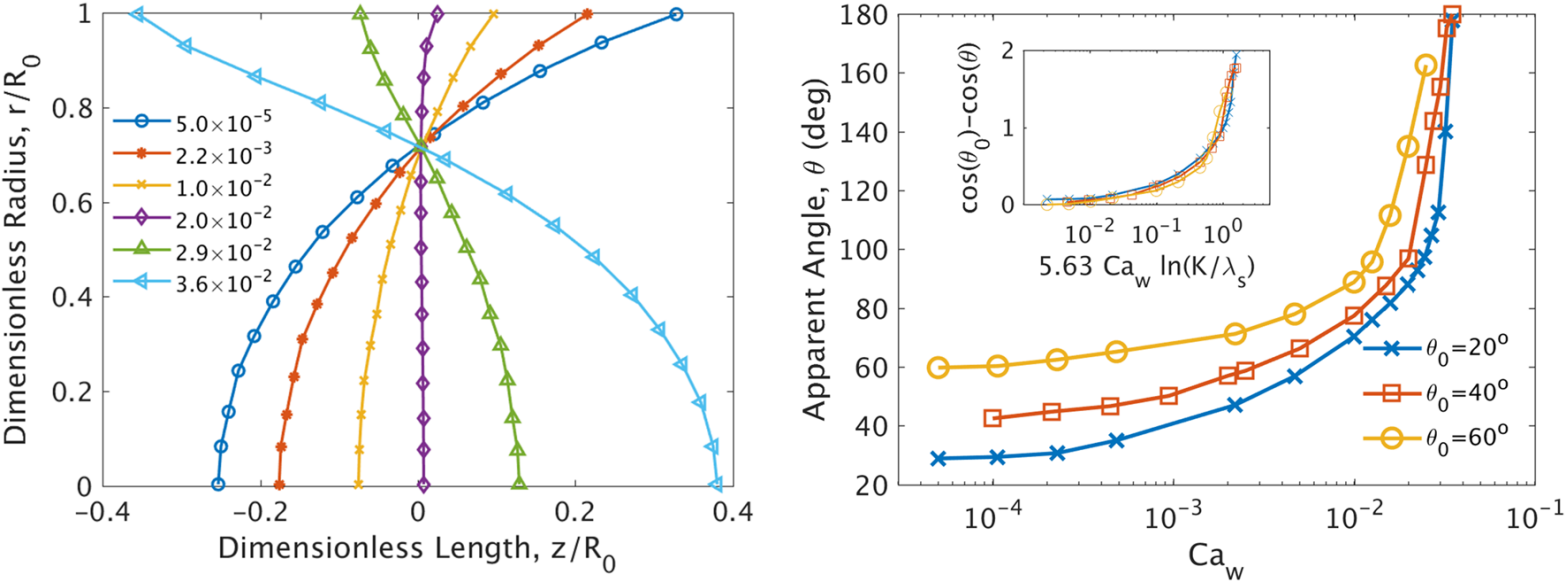
(Left) Fluid–fluid Interface shape for different values of the capillary number during imbibition in a single capillary with circular cross section. The interface is axisymmetric around the z-axis. $\theta _0=20$°. (Right) Calculated apparent contact angle vs $Ca_w$ of the meniscus. The curves correspond to 3 different equilibrium contact angles of 30, 40 and $60^{\circ }$. All curves converge asymptotically to their respective equilibrium value at low values of Ca, while they diverges as Ca increases above $Ca_w=10^{-2}$. It is very interesting to note the profound effect of $Ca_w$ on the the curvature of the interface that may even lead to a change in its concavity in favor of the non-wetting fluid at high $Ca_w$.

Figure 5 (right) shows the calculated contact angle for three different values of the initial equilibrium contact angle vs the Capillary number of the flow. The figure shows that all three curves reach asymptotically their corresponding equilibrium value when $Ca_w$ tends to zero, while they all diverge at $Ca_w \rightarrow 4\times 10^{-2}$. Interestingly enough, a simple rescaling proposed by Sheng and Zhou^59^ reveals that all curves can be described by a universal exponential function, *f*, by plotting all the data as follows;

14 $$\begin{aligned} cos(\theta _0)-cos(\theta )=f(5.63 Ca_w ln(K/\lambda _s^*)) \end{aligned}$$

where $\lambda _s^*=\lambda _s/R_0$ is the dimensionless slip length and the best fit is obtained for $K=0.3$ and $\lambda _s^*=10^{-4}$.

These results are in excellent agreement with those of Sheng and Zhou^59^, that were obtained however using a completely different numerical scheme, and further demonstrate the ability of the Level-Set model to capture complex interfacial dynamics during immiscible flows.

## Results and discussion

Following the above verification of our experimental setup and of the numerical model, we performed a series of drainage experiments within the PDMS micromodel presented in "Experimental microfluidic setup”. In these experiments dyed water (nw-phase) was injected at constant flow rate in the porous-like micromodel that was initially fully saturated by Fluorinert (w-phase), while recording the inlet pressure and the corresponding phase distribution patterns. The experiments were performed at 4 discrete values of the injection rate, i.e. $q=10^{-4}, 10^{-3}, 10^{-2}$ and $10^{-1}$ ml/min with corresponding water-based Reynolds numbers at the inlet channel equal to $Re=1.4\times 10^{-2}, 1.4\times 10^{-1}, 1.4\times 10^{0}$ and $1.4\times 10^{1}$. All the experiments were repeated several times to ensure the reproducibility of our results.

In parallel, we performed numerical simulations using the previously described Level-Set scheme in a 2D domain under comparable flow conditions. The effects of domain depth (z-dimension) were accounted for using an extra velocity dependent forcing term^45^ $\vec {F}_z=-\frac{12\mu }{b^2}\vec {u}$ which is added to right hand side of Eq. (1). This term corresponds to the solution of the momentum conservation equation for a Newtonian fluid during laminar flow between two parallel plates with distance *b* (here *b* = 115 $\mu m$). We should note here that while this approach is better-suited for creeping flow (i.e. $Re<1$), we adopt it here regardless of the actual value of *Re* in order to avoid extremely compute-intensive 3D simulations. We also assume that the radius of curvature at the fluid–fluid interface in the depth direction is constant and approximately equal to b/2 over the entire range of $Ca_w$ values in our study. This results in a fixed contribution to the overall capillary pressure, as we also discuss below.

### Capillarity-controlled regime

#### Experimental results

Starting with the lower injection flow rate ($q=10^{-4}$ ml/min), we postulate that in the limit of very low $Ca_w$ values (in the order of $10^{-6}$) the pressure drop due to viscous flow over the entire length of the microfluidic setup is practically negligible and the recorded inlet pressure readings correspond solely to the capillary pressure at fluid–fluid interface, $\Delta P_c$, as it advances through the pore-space. A common assumption in the limit of a Capillarity-controlled movement of the interface is that the radius of curvature of the interface that lies in a given pore-throat is proportional to the throat diameter, $D_t$, and the capillary pressure threshold for invading this throat is described by the Young–Laplace equation $\Delta P_c=2 \gamma cos(\theta _0) \left( \frac{1}{d} + \frac{1}{D_t} \right)$, where *d* is the fixed depth of the micromodel (here *d* = 115 $\mu m$). Therefore the recorded pressure should be correlated to the size of the currently invaded pore-throat.

**Figure 6:**
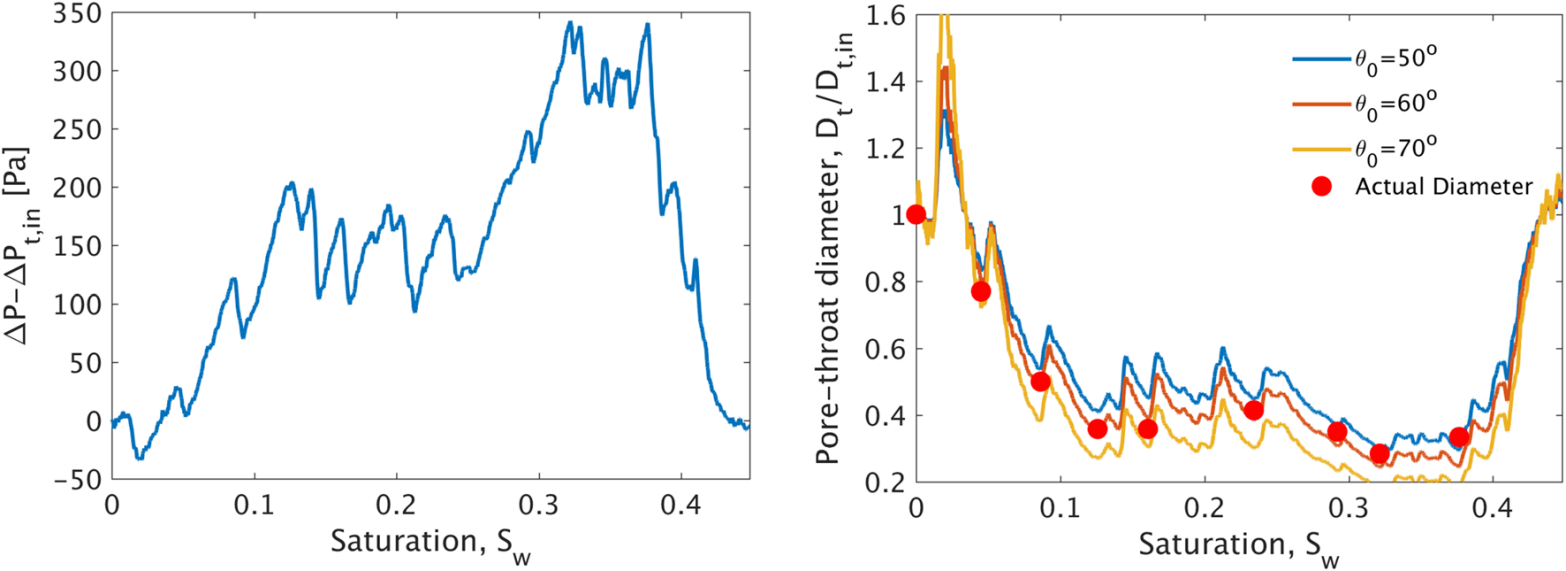
(Left) Experimental inlet pressure $\Delta P-\Delta P_{in}$ recorded during a drainage experiment in the capillarity-controlled regime (i.e. $q=10^{-4}$ ml/min and $Ca_w=1.5 \times 10^{-6}$. Here the fluid–fluid interface advances through a series of Haines jumps forming a single capillary finger. $\Delta P_{in}$ corresponds to the recorded pressure value when the interface first enters the patterned area of the micromodel from the inlet flow channel. (Right) Solution of the Young–Laplace equation for the throat diameter vs $S_w$ based on the recorded pressures for three values of the contact angle $\theta _0$. The actual throat diameters transversed by the interface at selected saturations values that correspond to local pressure maxima are shown as red circles.

We verify this assumption by plotting the recorded inlet pressure for $q=10^{-4}$ ml/min, which corresponds roughly to $Ca_w=1.5^{-6}$ (assuming $\theta _0\approx 55$ °), as a function of $S_w$, as shown in Fig. 6 (left). The pressure here is reported as $\Delta P-\Delta P_{in}$, where $\Delta P$ is the actual sensor reading and $\Delta P_{in}$ is the sensor reading that corresponds to the time when the interface first invaded through the inlet channel (i.e. $D_t$ = 500 $\mu m$). This serves for cancelling out any hydrostatic pressure effects related to the elevation of the sensor compared to the micromodel. Figure 6 (left) shows that the recorded pressure starts from zero and remains always positive until the interface reaches the outlet channel at $S_w>0.43$. Given that the diameter of the outlet channel is also $D_t$= 500 $\mu m$, the pressure reading becomes again equal to zero as expected. Furthermore, we postulate that the discrete pressure maxima of this curve correspond to pore-throat invasion events.

This is verified by visually inspecting the corresponding recorded phase distribution patterns (Supplementary Video 1). The video animation shows the dynamics of the invading aqueous phase (Left) and the corresponding inlet pressure reading as a red mark on the overall pressure plot (right). It demonstrates the actual pore-scale movement of the fluid–fluid interface that advances very slowly through the inlet channel and pore-bodies (i.e. larger spaces between several adjacent pillars) until it becomes apparently ‘trapped’ within pore-throats (i.e. minimum distance between two adjacent pillars). This apparent decrease in the advancing interface velocity is accompanied by a sharp increase in the recorded inlet pressure until the capillary pressure threshold is exceeded. Then the interface invades the pore-throat with the larger diameter and accelerates abruptly towards the adjacent pore-body. This sudden movement is known as a Haines jump^61^ and is commonly associated to Capillarity-controlled flow in porous media, where pore-bodies are invaded in a sequential fashion following the least-resistance pathway, as also shown in this case.

Based on these pressure measurements, it is also straightforward to recover the effective contact angle $\theta$ by correlating the recorded pressure dynamics to the known size, $D_t$, of the actual invaded pore-throat by a visual inspection of the interface movement. Assuming that the recorded pressure, $\Delta P$, is determined by the above equation plus a pressure offset, $P_0$, due to the calibration of the sensor and the hydrostatic pressure contribution within the inlet tubing, the recorded pressure is then;

15 $$\begin{aligned} \Delta P=\Delta P(D_t)=P_0+2 \gamma cos(\theta _0) \left( \frac{1}{d} + \frac{1}{D_t} \right) \end{aligned}$$

Here $P_0$ is constant throughout each drainage experiment, but may be somewhat different between experiments due to the slight movement of the tubings and intermediate reset of the pressure sensor. By assigning to the above equation a pore throat value of $D_{t,in}$ = 500 $\mu m$ corresponding to the recorded pressure sensor value $\Delta P_{in}$ when the interface passes through the inlet of the micromodel, and subtracting from $\Delta P$, the following correlation between $D_t$ and $\Delta P$ is derived;

16 $$\begin{aligned} D_t(\Delta P)=\frac{2\gamma cos(\theta _0) D_{t,in}}{2\gamma cos(\theta _0) +(\Delta P-\Delta P_{in})D_{t,in}} \end{aligned}$$

Given than $\Delta P=\Delta P(S_w)$, Fig. 6 (right) shows a plot of the above equation with respect to the corresponding value of $S_w$ for 3 different values of the wetting angle $\theta _0$. Also shown in red dots are the actual pore-throats diameters that are invaded by the interface at several saturation values. These values have been selected to correspond to local maxima in the $\Delta P$ vs $S_w$ curve. Our analysis reveals that the curve for $\theta _0=60$° best describes the actual pore throat diameter over the entire duration of the experiment. We will therefore use this value for the calculation of $Ca_w$ for the remaining of this manuscript.

**Figure 7:**
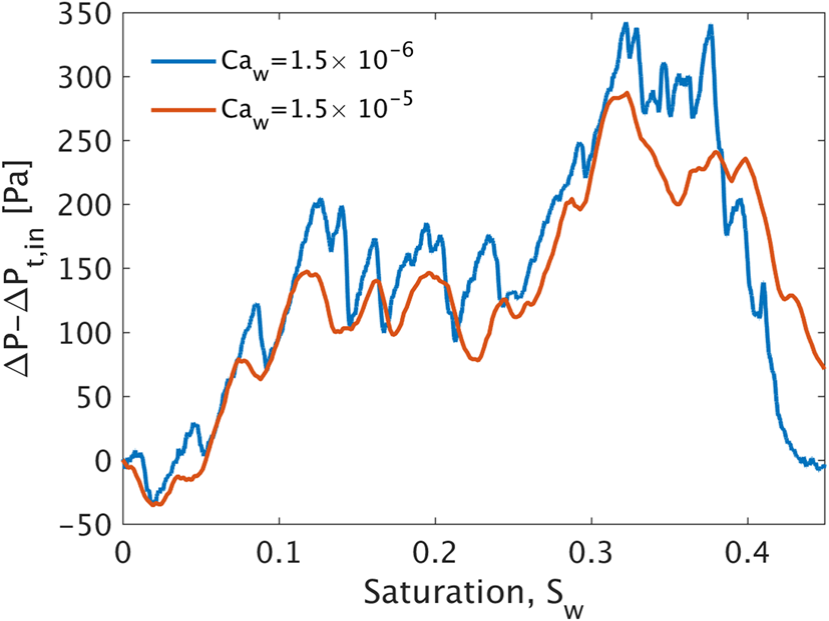
Experimental inlet pressure $\Delta P-\Delta P_{in}$ with respect to $S_w$ for $q=10^{-3}$ and $q=10^{-4}$ ml/min. Both curves exhibit local pressure maxima at practically identical saturations, thus revealing that fluid–fluid interface recedes following the same pore-throat pathway under the control of capillary forces.

We then increase the injection rate to $q=10^{-3}$ ml/min that corresponds to $Ca_w=1.5\times 10^{-5}$. It is very interesting to note in this case that the interface still invades one pore at a time in the form of Haines jumps^61^, following practically the same invasion pathway as above (see Supplementary Video 1 and Fig. 9). This demonstrates that capillary forces at the interface are still dominant over viscous ones (even after a tenfold increase in the injection flow rate), and thus the pore-scale scale geometry determines the invasion dynamics of the interface. This is also very well illustrated by comparing the recorded inlet pressure curves with respect to $S_w$ for the two cases as shown in Fig. 7. Despite a small shift towards lower pressures for the higher flow rate case, both curves exhibit a sequence of comparable pressure maxima at practically the same values of $S_w$ which eventually corresponds to the pressure ‘footprint’ of the invasion pathway (which is nearly identical for both values of *q*).

**Figure 8:**
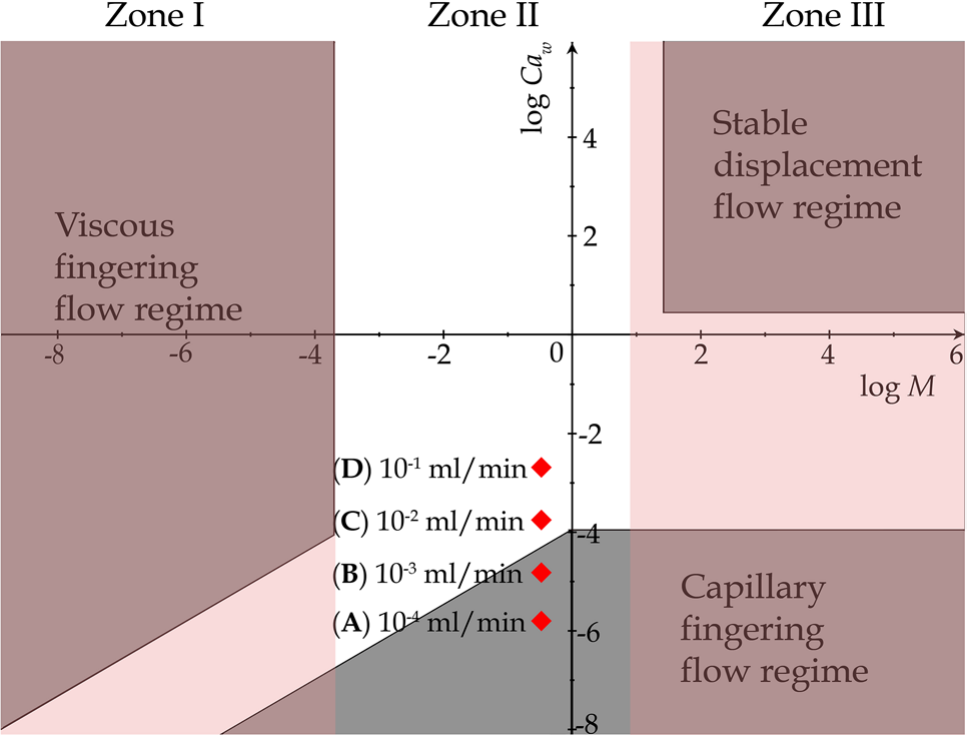
Phase diagram proposed by Lenormand et al^28^ that shows three distinct flow patterns (i.e. Viscous Fingering, Stable Displacement and Capillary Fingering) during immiscible flow in porous media as a function of $Ca_w$ and the viscosity ratio *M*. The position of our experiments are also marked in the diagram with red squares. Cases A and B lie clearly in the Capillary Fingering regime, while Cases C and D lie in the transitional zone where viscous forces become also important on interface movement.

Our results can be also discussed in the framework of the phase diagram proposed by Lenormand et al.^28^ for immiscible two-phase flows under different flow conditions, which was constructed based on elaborate pore-network simulations and displacement experiments in chemically etched micromodels. In Fig. 8 we reproduce the three distinct patterns proposed by Lenormand et al. (namely, Capillary Fingering, Stable Displacement and Viscous Fingering) with respect to the capillary number of the invading fluid, $Ca_w$ and the viscosity ratio *M*. We also highlight as points A and B the flow conditions that correspond to the previously reported experiments, that interestingly enough both lie in the Capillarity-controlled displacement region reported by Lenormand et al, and are thus expected to follow a similar invasion ‘pathway’ as determined solely by the pore-scale geometry of the micromodel.

#### Comparison between experimental and numerical results

**Figure 9:**
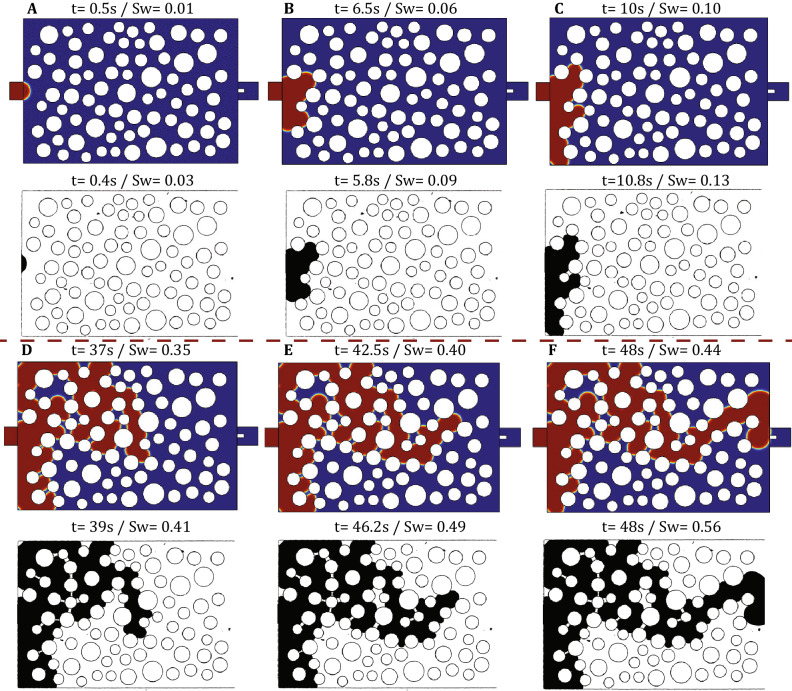
Comparison of the experimentally recorded phase distribution patterns with those of the numerical simulation for $q=10^{-3}$ ml/min ($Ca_w=1.5\times 10^{-5}$). In the numerical results, water is shown in red colour and Fluorinert in blue, while in the experimental snapshots dyed water is shown in black color and Fluorinert is transparent. The snapshots **(A–F)** correspond to identical locations of the most advanced downstream interface and the corresponding time and saturation are also reported above each figure.

We then proceed by comparing the experimental results for $q=10^{-3}$ ml/min to a numerical simulation under identical flow conditions. Figure 9 and Supplementary Video 2 show such a comparison for the experimental and numerical phase distribution patterns at discrete times and identical positions for the most-advanced edge of the fluid–fluid interface in the downstream direction. The comparison reveals that the numerical model captures quite well the dynamics of the invasion front over the entire range of $S_w$ (and t). Some minor differences in the invasion sequence can be observed very early in the simulation but these eventually only lead to a temporal shift that is clearly evident at breakthrough (namely, the invasion front reaches the outlet channel at $S_w=0.44$ at the numerical simulation and at $S_w=0.56$ at the experiment). Such differences could be due to impurities on surface the micro-structure of the micromodel during manufacturing that may effectively alter local wettability.

**Figure 10:**
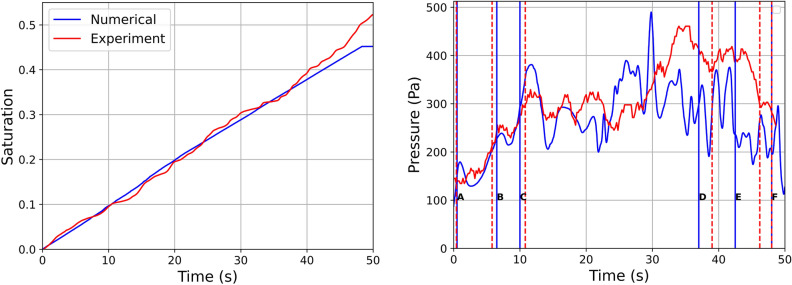
(Left) Comparison of saturation vs time for both numerical and physical experiments. (Right) Pressure drop comparison between physical and numerical experiments. The vertical coloured lines correspond to the flow patter comparison at Fig. 9.

We also show in Fig. 10 (left) the linear increase of the invading phase saturation, $S_w$, with respect to time, which is a clear demonstration of the applied constant injection rate by the microfluidic pump. Figure 10 (right) shows both the experimentally recorded inlet pressure (red curve) and the corresponding numerical results (blue curve) with respect to *t*. We can clearly verify that the match between the two curves is quite satisfactory during the first 15-20s, where both curves exhibit a series of local maxima that occur at practically identical times. This demonstrates that both experiment and simulation proceed through the same ‘pathway’ at early times (see Fig. 9A–C) and the corresponding pressure ‘footprint’ is quite accurately captured by both methods.

**Figure 11:**
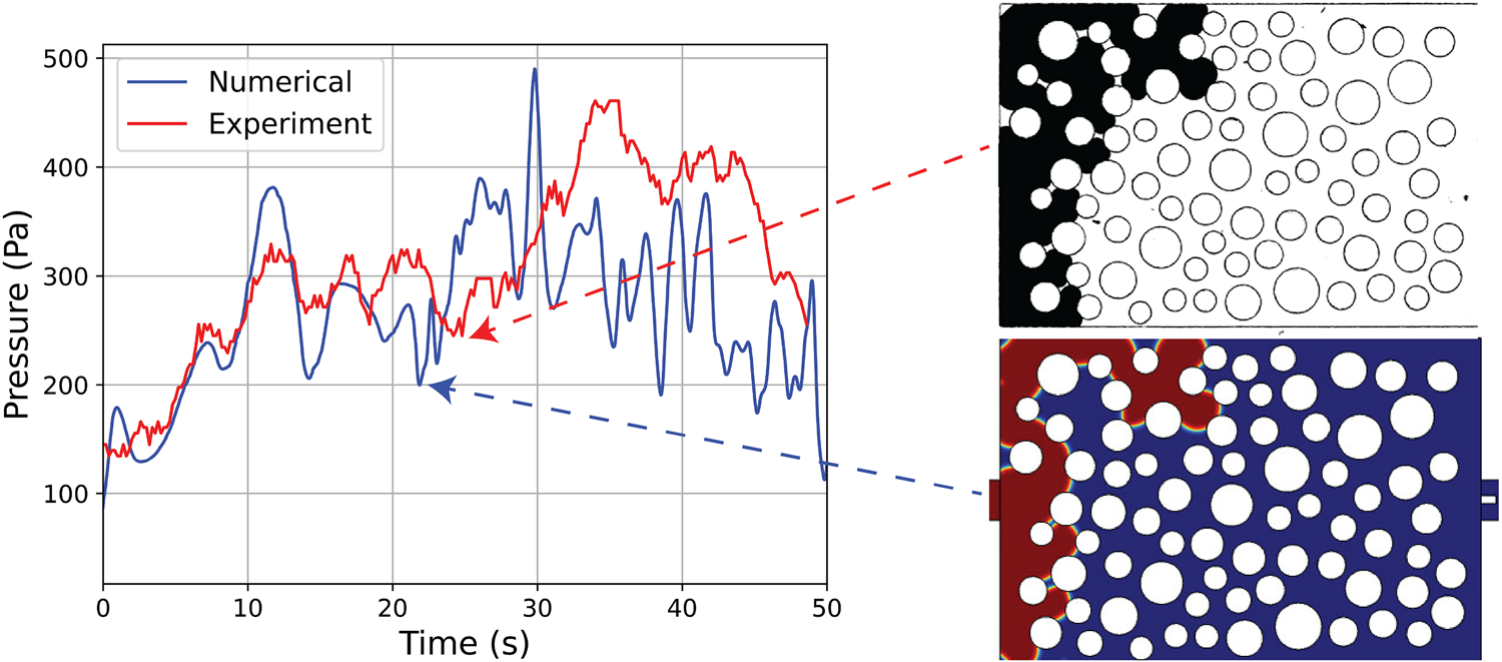
The change in flow pattern and its effect on pressure drop. The comparison between the physical and numerical experiments is in sync, until this change in flow pattern lead the changes in the pressure profile.

At later times however (after 20 s), some minor differences in the pore invasion sequence lead to different pressure dynamics. These are visible in Fig. 9D–F) and are also manifasted in the pressure footprint of Fig. 10 (right) as a temporal shift between the two curves. This shift is shown in more detail in Fig. 11 where a minor mismatch in the pore invasion sequence (at the top left of domain) leads to a shift in the pressure profiles between experimental and numerical results. Such discrepancies are also responsible for the larger value of $S_w$ at breakthrough in the experiment compared to the numerical simulation. Despite this fact, we should note that both curves exhibit practically the same range of pressures in the range of 150 to 450 Pa, thus revealing that both approaches accurately capture the available range of capillary pressure thresholds associated with the spatial pore-throat distribution.

### Onset of viscosity dominant regime

**Figure 12:**
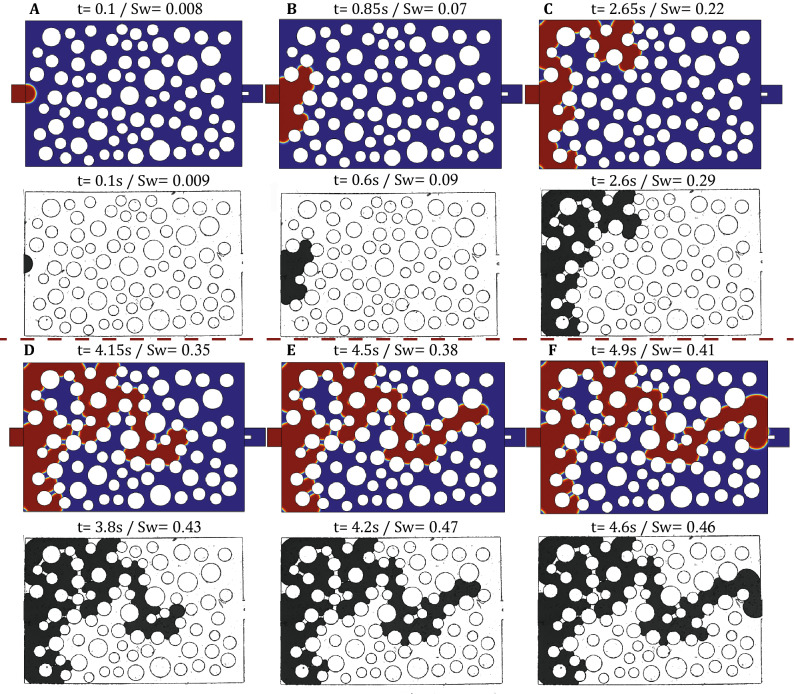
Comparison of the experimentally recorded phase distribution patterns with those of the numerical simulation for $q=10^{-2}$ ml/min ($Ca_w=1.5\times 10^{-4}$). In the numerical results, water is shown in red colour and Fluorinert in blue, while in the experimental snapshots dyed water is shown in black color and Fluorinert is transparent. The snapshots **(A–F)** correspond to identical locations of the most advanced downstream interface and the corresponding time and saturation are also reported above each figure.

A further increase of the injection flow rate at $q=10^{-2}$ ml/min ($Ca_w=1.5\times 10^{-4}$) is expected to lead to a significant viscosity-induced pressure drop both along inlet/outlet flow channels and the porous-like section of the micromodel. In this case we found that the sampling frequency of the pressure sensor is not sufficiently high to adequately reflect the topology and the movement of the interface within the porous domain. However, the relative effects of viscosity over capillary forces on interfacial dynamics can still be recorded visually using the open-air microscope of our experimental setup. Figure 12 shows the experimentally recorded phase distribution patterns and the corresponding numerical simulation results at a sequence of times for $q=10^{-2}$ ml/min. These figures reveal once again both a satisfactory match of the numerical simulation with the experiment results, but also demonstrate that the invasion sequence still remains capillarity-controlled (similar to $q=10^{-4}$ and $q=10^{-3}$ ml/min). This result is by no means self-evident given that the pore invasion sequence is found to remain persistently stable over 3 orders of magnitude for the imposed injection flow rate, while the duration of the invasion experiment decreases significantly from approximately 500s (for $q=10^{-4}$ ml/min) to 5s (for $q=10^{-2}$ ml/min).

To interpret these findings we return on the phase diagram of Fig. 8 where the previously reported experimental conditions are highlighted as (A), (B) and (C). While experiments (A) and (B) clearly lie in the Capillary Fingering zone, experiment (C) is located outside this zone but very close to its boundary with the intermediate transitional area between the three different flow domains. The transional area identified by Lenormand et al demonstates the combination of flow conditions where viscous and capillary forces become equally important on the movement of the interface. We would therefore expect a divergence from the above invasion sequence for flow conditions that lie within this region.

By increasing the flow rate even more at $q=10\times ^{-1}$ ml/min (Point (D) in Fig. 8), we observe for the first time a clear deviation from the capillarity controlled regime as shown in Supplementary Video 3. In this case the front advances by invading several pores simultaneously in the form of 2-3 ‘viscous’ fingers that move at comparable velocities. The reproducibility of these results were using a series of experiments under the same flow conditions, revealing only minor pore-invasion differences between each realization. Apparently, the flow conditions now lie clearly in the transitional area of the Lenormand phase diagram, where the pore throat invasion criterion is satisfied for several pore-throats along the current position of the interface simultaneously. Given that the viscosity-induced pressure gradient within the receding Fluorinert phase is practically 5 times larger than in the advancing aqueous phase (assuming a fixed displacement velocity), we would expect an unstable displacement (as opposed to a stable piston-like front) where multiple viscous fingers form simultaneously. This situation is quite different than experiments (A)–(C) where the viscous pressure gradient is negligible at a length scale comparable to the domain size compared to Capillary pressure (e.g. at a length scale equal to L = 6mm), while in experiment (D) the viscous pressure gradient is significant at length scale of the pore size (e.g. at a length scale equal to $D_t$).

**Figure 13:**
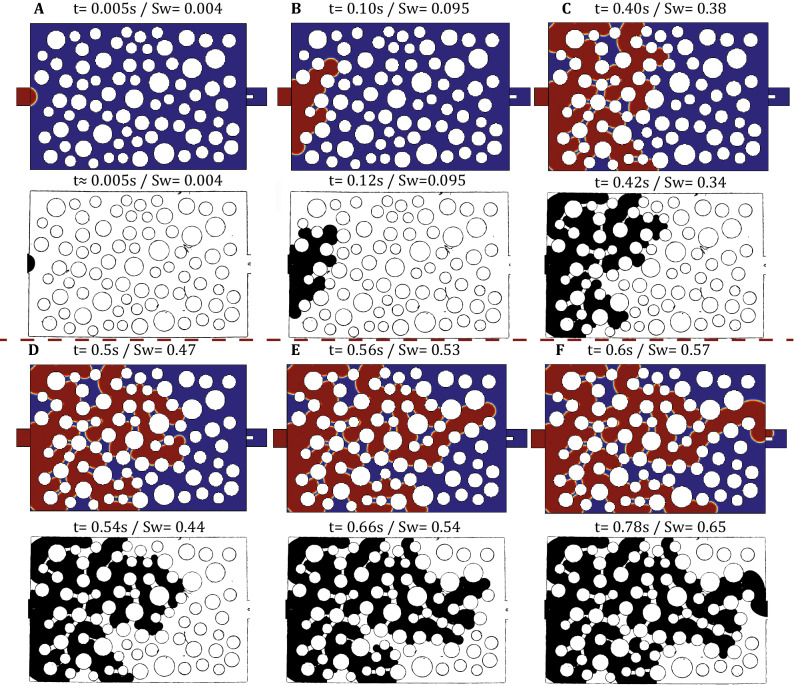
Comparison of the experimentally recorded phase distribution patterns with those of the numerical simulation for $q=10^{-1}$ ml/min ($Ca_w=1.5\times 10^{-3}$). In the numerical results, water is shown in red colour and Fluorinert in blue, while in the experimental snapshots dyed water is shown in black colour and Fluorinert is transparent. The snapshots **(A–F)** correspond to identical locations of the most advanced downstream interface and the corresponding time and saturation are also reported above each figure.

A comparison between experiment and a numerical simulation for $q=10^{-1}$ ml/min ($Ca_w=1.5\times 10^{-3}$) is shown in Fig. 13 and Supplementary Video 3. Even at these extreme flow conditions and despite the simplified 2D numerical approach, we conclude that our numerical model captures quite well the dynamics of the invasion process (at least during earlier times as previously) both in terms of phase distribution patterns but also in terms of the actual invasion time (which less than 1s under these conditions). At later times, the simultaneous invasion of multiple viscous fingers competing to occupy the available pore space leads to some differences between experiment and numerical simulation. This competition also results in a better sweeping efficiency (0.78 compared to 0.46 for $q=10^{-2}$ ml/min) in our microfluidic domain that has a finite width. However, this result may be misleading given that all fingers emanating from the same single inlet channel are reflected (redirected) back to the porous domain when they reach the side walls. We thus argue that finite size and wall effects are more important in this viscosity-dominated case than in the previous capillarity-controlled experiments.

## Conclusions

In this work we propose a novel microfluidic setup capable of monitoring simultaneously the inlet pressure and corresponding phase distribution patterns during immiscible displacement in an artificial porous-like PDMS domain. We demonstrate that our experimental setup and high sensitivity pressure sensor is capable of resolving both capillary and viscous pressure drop across both in PTFE capillaries and provide results comparable to the Washburn model. By injecting a non-wetting fluid at fixed flow rates into the PDMS domain that consists of randomly distributed pillars, initially saturated by a wetting fluid, we are able to accurately correlate the inlet pressure with the dynamics of the fluid–fluid interface as it recedes through the pore space over a wide range of Capillary numbers that spans 4 orders of magnitude. Our results reveal that in the low Ca limit, the movement of the interface is controlled by capillary forces that develop within pore-throats and the effects of viscosity are negligible. At higher Ca values, however, we are able to identify the onset of a viscosity-controlled flow regime, where multiple pore-throats are across the interface are invaded simultaneously due to combined effects of viscous and capillary forces at the pore-scale. Our experimental results are then compared with numerical simulations obtained using a robust Level-Set capable of resolving interfacial dynamics are extremely fine sub-pore resolutions. The model is found to recover quite satisfactory the phase distributions patterns over the entire range of Ca values (both in the capillary and the viscous-dominated flow regimes), while it predicts the corresponding inlet dynamics quite clearly at lower Ca values.

## Supplementary information

Supplementary Video 1.

Supplementary Video 2.

Supplementary Video 3.
