# Author Correction: Pore-scale effects during the transition from capillary to viscosity-dominated flow dynamics within microfluidic porous-like domains

**DOI:** 10.1038/s41598-021-00842-1

**Published:** 2021-10-27

**Authors:** A. Yiotis, N. K. Karadimitriou, I. Zarikos, H. Steeb

**Affiliations:** 1 School of Mineral Resources Engineering, Technical University of Crete, Chania, Greece; 2 Institute of Mechanics (CE), University of Stuttgart, Stuttgart, Germany; 3 Environmental Research Laboratory, National Center for Scientific Research ‘Demokritos’, Agia Paraskevi, Greece; 4 Stuttgart Center for Simulation Technology, University of Stuttgart, Stuttgart, Germany

Correction to: *Scientific Reports* 10.1038/s41598-021-83065-8, published online 16 February 2021

The original version of this Article contained errors.

In the Materials and Methods section under the subheading ‘Experimental microfluidic setup’,

“As shown in Figure 1a, the micromodel consists of a central rectangular area 6 mm long and 4 mm wide and two flow channels (inlet and outlet) both 13 mm long and 500 *μm* wide.”

now reads:

“As shown in Fig. 1 (top), the micromodel consists of a central rectangular area 6 mm long and 4 mm wide and two flow channels (inlet and outlet) both 13 mm long and 500 *μm* wide.”

“The entire micromodel is 115 *μ*m deep and the rectangular central area contains 76 non-overlapping cylindrical pillars (that appear as circles in Figure 1b with diameters selected from a random size distribution in the range of 275 and 575 *μm* (average diameter of 380 *μm*), as shown in Fig. 1c.”

now reads:

“The entire micromodel is 115 *μm* deep and the rectangular central area contains 76 non-overlapping cylindrical pillars (that appear as circles in Fig. 1 (left) with diameters selected from a random size distribution in the range of 275 and 575 *μm* (average diameter of 380 *μm*), as shown in Fig. 1 (right).”

Additionally, in the Materials and Methods section under the subheading ‘Immiscible flow in a single capillary at low Ca values’,

$$ \frac{{Ca_{w} }}{\xi } \ll 1\; then \frac{{\Delta P\left( {S_{w} } \right)}}{{\Delta P_{c} }} = 4 \frac{{Ca_{w} }}{\xi }\left( {\frac{{\mu_{o} - \mu_{o} }}{{\mu_{o} }}S_{w} + \frac{{\mu_{o} }}{{\mu_{o} }}} \right) $$

now reads:

$$ \frac{{Ca_{w} }}{\xi } \gg 1\; then \frac{{\Delta P\left( {S_{w} } \right)}}{{\Delta P_{c} }} = 4 \frac{{Ca_{w} }}{\xi }\left( {\frac{{\mu_{o} - \mu_{o} }}{{\mu_{o} }}S_{w} + \frac{{\mu_{o} }}{{\mu_{o} }}} \right) $$

The original Article has been corrected.

